# γ-Aminobutyric acids (GABA) and serum GABA/AABA (G/A) ratio as potential biomarkers of physical performance and aging

**DOI:** 10.1038/s41598-023-41628-x

**Published:** 2023-10-10

**Authors:** Charalampos Lyssikatos, Zhiying Wang, Ziyue Liu, Stuart J. Warden, Lynda Bonewald, Marco Brotto

**Affiliations:** 1 Indiana Center for Musculoskeletal Health, Indiana University School of Medicine, Indianapolis, IN USA; 2 Bone-Muscle Research Center, College of Nursing and Health Innovation, University of Texas-Arlington, 655 W. Mitchell Street, Science-Engineering-Innovation Research (SEIR) Suite 272, Arlington, TX 76010 USA; 3 Department of Biostatistics and Health Data Science, Indiana University School of Medicine, Indianapolis, IN USA; 4 Department of Physical Therapy, School of Health and Human Sciences, Indiana University, Indianapolis, IN USA

**Keywords:** Physiology, Medical research

## Abstract

Declining physical performance with age and disease is an important indicator of declining health. Biomarkers that identify declining physical performance would be useful in predicting treatment outcomes and identifying potential therapeutics. γ-aminobutyric acid (GABA), a muscle autocrine factor, is a potent inhibitor of muscle function and works as a muscle relaxant. *L*-α-aminobutyric acid (*L*-AABA) is a biomarker for malnutrition, liver damage, and depression. We sought to determine if GABA and *L*-AABA may be useful for predicting physical performance. Serum levels of GABA and *L-*AABA were quantified in 120 individuals divided by age, sex, and physical capacity into low, average, and high performer groups. Analyses explored correlations between serum levels and physical performance. Both GABA and the ratio of GABA/AABA (G/A), but not AABA, were highly positively associated with age (Pearson correlations *r* = 0.35, *p* = 0.0001 for GABA, *r* = 0.31, *p* = 0.0007 for G/A, n = 120). GABA showed negative associations in the whole cohort with physical performance [fast gait speed, 6 min walk test (6MWT), PROMIS score, and SF36PFS raw score] and with subtotal and femoral neck bone mineral density. *L*-AABA was positively associated with usual gait speed, 6MWT, total SPPB score, and SF36PFS raw score in the total cohort of 120 human subjects, also with 6MWT and SF36PFS raw score in the 60 male subjects, but no associations were observed in the 60 females. As both GABA and *L*-AABA appear to be indicative of physical performance, but in opposite directions, we examined the G/A ratio. Unlike GABA, the G/A ratio showed a more distinct association with mobility tests such as total SPPB score, usual and fast gait speed, 6MWT, and SF36PFS raw score in the males, regardless of age and metabolic status. Serum G/A ratio could be potentially linked to physical performance in the male population. Our findings strongly suggest that GABA, *L*-AABA, and the G/A ratio in human serum may be useful markers for both age and physical function. These new biomarkers may significantly enhance the goal of identifying universal biomarkers to accurately predict physical performance and the beneficial effects of exercise training for older adults.

## Introduction

In the United States with the increase of life expectancy, the population with an age above 65 will be 20–25% of the total population by 2050^1^. Aging is believed to be a combination of body deterioration and disease^2^. The gradual process of bodily deterioration, inevitable changes in cellular structure and function that takes place throughout life is known as primary aging. Secondary aging is the process resulting from disease, lifestyle and environmental influences, and is often preventable through lifestyle choice or modern medicine^2^. Aging has deleterious effects on muscle and bone, thus reducing physical performance^3^ and increasing frailty, risk of fall and fractures, leading to chronic disability to the elderly population^4^. The human body consists of more than 500 muscles, regulated by the nervous system to interact with the skeleton^5^. The synergistic actions of muscles are responsible for physical activity resulting in energy consumption^6^ and is well-established as a countermeasure against secondary aging^7^. Exercise has many beneficial effects on the cardiorespiratory system and muscle mass/function by preventing age-associated insulin resistance and diminished mitochondrial capacity in skeletal muscle^8^. However, the mechanisms behind this beneficial effect on musculoskeletal health are still unclear. Therefore, there is an unmet need for biomarkers to detect the effects of physical activity on muscle performance in order to identify new strategies to achieve healthy aging.

Biomarkers have been postulated as essential variables to measure the effects of physical activity on the human body. Generally, these biomarkers are secreted molecules called myokines or cytokines related with performance (muscle status, endocrine response, and oxygen transport), health (nutritional and hydration status, allergies), and recovery (inflammation, injury status and risk, muscle damage)^9^. Although some published studies have found associations between physical activity, performance, and biomarkers in cohorts with different age, race, and physiological or pathological conditions^10,10–13^, there is still a need for additional universal biomarkers, useful for generalized populations. For example, a published study performed in a cross-sectional population-based sample reported that physical activity and performance are associated with lower levels of inflammatory biomarkers (C-reactive protein, interleukin-6, and fibrinogen) in plasma from the elderly population (aged 65 years or older)^13^. However, only the 400-m walking test was used in that study as a performance-based measurement, and the levels of physical activity were classified based on self-reported information which limits the validity and strength of the study. In the present study, we use multiple tests not self-reported.

Aminobutyric acids are nonproteinogenic amino acids which may be released during physical activity and impact physical performance. They comprise three types of isomers: α-aminobutyric acid (2-aminobutyric acid, AABA), β-aminobutyric acid (3-aminobutanoic acid, BABA), β-aminoisobutyric acid (3-aminoisobutyric acid, BAIBA), and γ-aminobutyric acid (4-aminobutyric acid, GABA). Except for GABA, each isomeric aminobutyric acid has two mirror-image enantiomers with the same molecular structures but significantly different biological functions. Our research laboratory has developed a fast and sensitive method for complete separation and accurate measurement of the aminobutyric acids in biological samples using liquid chromatography with tandem mass spectrometry (LC–MS/MS)^14^.

AABA has been postulated to have antioxidant activity and found to be a biomarker for alcohol-induced liver injury, sepsis, malnutrition, and depression^15,16,16,18^. *L*-AABA was further reported as the only identified enantiomer in human and mouse biological fluid samples including serum, plasma, and cerebrospinal fluid, and showed significant association with expression levels of *UPB1*, a gene encoding β-ureidopropionase belonging to the CN hydrolase family with significant association with bone mineral density (BMD)^14^. GABA is present in bacteria where it is functionally involved in spore germination, and it also confers resistance to acidic pH to several bacteria^17^, in fungi where it works as a source of carbon and nitrogen^21^, and in plants where it plays key roles in growth and development^18^. In humans, it is a major inhibitory neurotransmitter in the central nervous system that regulates internal neuronal communication but also acts as a muscle relaxant during sleep and in setting overall muscle tone^19^. It is also an antioxidant and anti-inflammatory amino acid^20^. Our previous studies have shown that GABA has potential to serve as biomarker for post-menopausal osteoporosis^14^.

In this study, serum concentrations of GABA, *L-*AABA, and their ratio were quantified from 120 healthy non-Hispanic human subjects (aged between 20 and 85 years) with completed information of physical characteristics and performance. We identified associations between serum levels and physical performance assessments, and further investigated the potential of these aminobutyric acids to be used as biomarkers of physical performance and age. These markers can also potentially serve as the cornerstone to investigate new therapies for aging-related musculoskeletal diseases.

## Methods

### Recruitment of human subjects

Serum samples and data were obtained from 120 individuals who had visited the Musculoskeletal Function, Imaging, and Tissue Resource Core (FIT Core) of the Indiana Center for Musculoskeletal Health’s Clinical Research Center (Indianapolis, Indiana) between 3/2018 and 4/2019. The FIT Core serves to provide: (1) standardized performance of physical function tests and patient reported outcomes related to physical function, (2) imaging outcomes for body composition and bone health, and (3) the collection and banking of biological samples within the Indiana Biobank.

Participants were recruited to the FIT Core by self-referral from the local community and by investigators seeking outcomes related to musculoskeletal health for their research subjects. The Core has Institutional Review Board approval from Indiana University (IU–IRB) for sample and data collection and storage from all-comers who provide written informed consent, irrespective of current or previous health status. All methods were approved by the IU–IRB and performed in accordance with the Declaration of Helsinki and the relevant guidelines and regulations of the IU–IRB. The consent provided by participants allows for deidentified samples and data to be stored and retrieved. Additional approval was obtained from the IU-IRB for the current analyses.

The FIT Core collected samples and data from 1518 individuals between 3/2018 and 4/2019. To be included in the current analyses, individuals needed to be 20–85 years of age, self-reported white and non-Hispanic, and without a self-reported major chronic disease. Individuals within each sex were stratified into four age groups (20–34, 35–49, 50–64, and 65 + yrs) and ranked for their performance on the FIT Core’s hand grip strength test and test of the number of chair stands completed in 30 s. These tests were selected as they assess function of the upper and lower extremities, are predictive of poor outcomes, and are the two skeletal muscle strength tests recommended for identifying sarcopenia^21^. The age-and sex-specific rankings on each of the two tests were summed within each individual to create a single composite ranking. The 5 individuals within each sex and age group with the worst, average, and best composite rank were selected and grouped as low (LP), average (AP), and high (HP) performers, respectively (Table 1). The detailed characteristics of the study subjects shown in Supplementary Table 1.

**Table 1:** Characteristics on females and males by performance (low, average, high).

	20–34 years			35–49 years			50–64 years			65 +		
	Low	Average	High	Low	Average	High	Low	Average	High	Low	Average	High
Females												
Age (yr)	26.6 ± 3.8	27.4 ± 4.2	27.5 ± 5.2	44.3 ± 5.5	45.2 ± 3.5	45.1 ± 5.1	57.2 ± 4.4	57.9 ± 2.7	53.3 ± 1.3	76.4 ± 7.1	73.9 ± 1.6	68.3 ± 3.5
Height (m)	1.62 ± 0.07	1.68 ± 0.05	1.67 ± 0.05	1.63 ± 0.05	1.65 ± 0.06	1.69 ± 0.05	1.63 ± 0.02	1.64 ± 0.06	1.69 ± 0.08	1.62 ± 0.08	1.59 ± 0.04	1.64 ± 0.04
Weight (kg)	67.4 ± 10.3	63.1 ± 5.2	69.0 ± 6.7	84.7 ± 13.1	73.2 ± 9.6	75.2 ± 14.0	76.7 ± 12.5	72.9 ± 10.0	72.7 ± 12.2	71.6 ± 13.6	65.7 ± 10.5	71.4 ± 23.9
BMI (kg/m^2^)	25.6 ± 3.9	22.5 ± 1.6	25.0 ± 3.4	31.8 ± 5.4	27.1 ± 4.8	26.5 ± 5.1	29.0 ± 5.3	27.3 ± 4.4	25.7 ± 3.8	27.3 ± 3.9	26.1 ± 3.8	26.9 ± 10.3
PROMIS physical function score	54.2 ± 7.1	62.0 ± 6.9	63.0 ± 4.4	52.2 ± 3.3	51.3 ± 3.8	57.9 ± 8.9	53.1 ± 6.9	51.4 ± 5.0	64.9 ± 5.7	48.3 ± 2.5	53.1 ± 4.0	54.6 ± 3.82
SF36-physical function score	96 ± 4	100 ± 0	100 ± 0	96 ± 4	93 ± 8	96 ± 5	92 ± 8	87 ± 6	99 ± 2	87 ± 12	85 ± 9	95 ± 6
Grip strength (kg)	17.4 ± 3.1	28.2 ± 0.9	37.0 ± 2.5	16.9 ± 6.5	26.8 ± 0.6	37.1 ± 2.6	16.7 ± 2.4	25.4 ± 0.3	35.8 ± 2.1	17.1 ± 4.0	21.5 ± 0.8	31.2 ± 3.0
Grip strength (z-score)	−1.72 ± 0.52	0.04 ± 0.13	1.43 ± 0.35	−1.73 ± 1.17	0.10 ± 0.15	1.90 ± 0.39	−1.53 ± 0.56	0.16 ± 0.13	1.98 ± 0.41	−0.74 ± 1.24	0.09 ± 0.16	1.91 ± 0.67
Repeat chair stands in 30 s (n)	12.4 ± 2.2	17.8 ± 1.3	24.6 ± 3.1	11.4 ± 0.6	16.8 ± 0.8	25.6 ± 3.8	11.8 ± 0.8	15.0 ± 1.0	22.4 ± 2.1	11.4 ± 2.1	13.2 ± 1.3	19.8 ± 1.8
Repeat chair stands in 30 s (z-score)	−1.55 ± 0.81	0.01 ± 0.37	1.53 ± 0.51	−1.56 ± 0.24	0.11 ± 0.21	1.87 ± 0.61	−1.16 ± 0.24	−0.10 ± 0.27	1.50 ± 0.42	−0.67 ± 0.65	−0.06 ± 0.49	1.55 ± 0.48
**Males**												
Age (yr)	23.9 ± 4.5	26.4 ± 3.1	26.9 ± 4.6	40.6 ± 2.2	40.9 ± 3.8	42.7 ± 5.5	62.2 ± 3.1	57.9 ± 5.5	54.1 ± 3.8	73.9 ± 6.6	70.5 ± 6.3	69.0 ± 5.5
Height (m)	1.77 ± 0.08	1.80 ± 0.06	1.82 ± 0.06	1.82 ± 0.05	1.74 ± 0.05	1.77 ± 0.07	1.77 ± 0.10	1.80 ± 0.05	1.79 ± 0.11	1.70 ± 0.04	1.74 ± 0.05	1.75 ± 0.04
Weight (kg)	71.0 ± 9.0	90.5 ± 15.0	82.8 ± 10.1	84.9 ± 11.8	87.9 ± 14.5	84.1 ± 10.3	80.1 ± 3.5	91.1 ± 13.5	87.7 ± 12.9	88.7 ± 14.8	80.3 ± 9.8	82.1 ± 8.4
BMI (kg/m^2^)	22.9 ± 2.0	27.9 ± 4.6	24.9 ± 2.8	25.5 ± 3.3	29.1 ± 4.9	26.8 ± 3.1	26.8 ± 2.6	28.0 ± 3.2	27.4 ± 2.3	30.8 ± 6.5	26.5 ± 3.4	26.8 ± 3.1
PROMIS physical function score	56.2 ± 6.7	59.9 ± 5.6	66.2 ± 4.7	56.1 ± 9.5	58.1 ± 4.2	67.8 ± 1.5	54.3 ± 7.0	53.1 ± 1.3	58.6 ± 5.4	47.7 ± 6.3	55.7 ± 5.4	56.2 ± 6.4
SF36-physical function score	97 ± 4	99 ± 2	100 ± 0	95 ± 5	99 ± 6	100 ± 0	92 ± 8	97 ± 3	98 ± 3	51 ± 29	87 ± 19	93 ± 10
Grip strength (kg)	34.5 ± 3.7	47.8 ± 1.3	59.0 ± 3.2	40.3 ± 2.1	48.1 ± 1.2	56.5 ± 3.6	34.6 ± 3.8	43.1 ± 3.1	52.6 ± 6.0	23.0 ± 7.9	36.5 ± 1.4	41.8 ± 1.8
Grip strength (z-score)	−1.26 ± 0.34	0.03 ± 0.18	1.03 ± 0.24	−0.72 ± 0.22	0.06 ± 0.33	0.88 ± 0.27	−0.58 ± 0.51	0.21 ± 0.38	1.05 ± 0.67	−1.49 ± 0.95	0.13 ± 0.55	0.73 ± 0.24
Repeat chair stands in 30 s (n)	12.0 ± 1.9	18.2 ± 1.5	26.6 ± 3.9	15.4 ± 0.9	19.2 ± 3.4	26.0 ± 3.8	13.4 ± 2.9	16.8 ± 1.1	21.8 ± 2.9	9.8 ± 1.9	14.8 ± 2.5	21.6 ± 2.3
Repeat chair stands in 30 s (z-score)	−1.79 ± 0.72	0.04 ± 0.36	1.72 ± 0.61	−0.55 ± 0.22	0.33 ± 0.66	1.62 ± 0.62	−0.63 ± 0.80	0.14 ± 0.27	1.06 ± 0.49	−1.26 ± 0.63	0.02 ± 0.62	1.38 ± 0.56

### Physical function

The FIT Core assessed dominant hand grip strength (Jamar Plus + digital hand dynamometer; Sammons Preston, Bolingbrook, IL), number of chair stands completed in 30 s, and time taken to complete 5 chair stands, as we have previously described^22^. In addition to raw values, grip strength and repeat chair stand outcomes were converted to age- and sex-matched *z* scores relative to reference data obtained in the FIT Core^22^. Time to walk 4-m from a stationary start at normal speed (usual gait speed) and as quickly as possible without running (fast gait speed) were measured with a stopwatch and converted to speed (m/s), as we have previously reported^27^.

Results from the repeat chair stand, usual gait speed, and a static balance test (ability balance for 10 s with feet side-by-side, semi-tandem, and tandem) were used to calculate the Short Physical Performance Battery (SPPB) score out of 12^23^. Distance walked in 6 min was measured according to the American Thoracic Society^24^. The physical function (PF) domain of the NIH Patient Reported Outcomes Measurement Information System (PROMIS) computerized adaptive test (CAT) (PROMIS-CAT-PF) (version 1.2) and the physical functioning subscale of the Short Form 36 (SF-36 PF) were used to assess self-reported functional health.

### Body composition and bone health

Participant height (to nearest 0.1 cm) and mass (to nearest 0.1 kg) were measured without shoes using a calibrated stadiometer (Seca 264; Seca GmbH & Co., Hamburg, Germany) and scale (MS140-300; Brecknell, Fairmont, MN), respectively. Body mass index (BMI; kg/m^2^) was calculated as body mass relative to height squared. Appendicular lean mass relative to height squared (kg/m^2^) and whole-body BMD, fat mass, percent were assessed by whole-body dual-energy x-ray absorptiometry (DXA) (Norland Elite; Norland at Swissray, Fort Atkinson, WI). Regional DXA using the same scanner assessed hip and spine BMD.

### Chemicals and reagents

Aminobutyric acid standard compounds (S)-2-aminobutyric acid (*L*-AABA) and (R)-2-aminobutyric acid (*D*-AABA) were purchased from Thermo Fisher Scientific (Waltham, MA), and 4-aminobutyric acid (GABA) was purchased from Sigma-Aldrich (St. Louis, MO). Isotopic internal standard (IS) compounds *DL*-2-aminobutyric-2,3,3,4,4,4-d_6_ acid (*D,L*-AABA-d_6_) and ( ±)-3-amino-iso-butyric-2,3,3-d_3_ acid (*D,L*-BAIBA-d_3_) were obtained from CDN Isotopes (Pointe-Claire, Quebec, Canada), and 4-aminobutyric-d_6_ acid (GABA-d_6_) was purchased from Sigma-Aldrich (St. Louis, MO). Formic acid (reagent grade, ≥ 95%), Bovine Serum Albumin (BSA) were obtained from Sigma–Aldrich (St. Louis, MO). Phosphate Buffered Saline (PBS) was purchased from Fisher Scientific (Pittsburgh, PA). HPLC–MS grade acetonitrile, water, and methanol were purchased from J.T. Baker (Phillipsburg, NJ).

### LC–MS/MS conditions

All components of LC–MS/MS system are from Shimadzu Scientific Instruments, Inc. (Columbia, MD). the LC system was equipped with pumps A and B (LC-30AD), and autosampler (SIL-30AC). The LC separation was conducted on a chiral SPP-TeicoShell column (150 × 4.6 mm, 2.7 µm, AZYP LLC., Arlington, TX) configured with a Synergi™ 4 µm Max-RP column as guard column (50 × 2.0 mm, Phenomenex, Torrance, CA). The MS/MS analysis was performed on Shimadzu LCMS-8050 triple quadrupole mass spectrometer.

Quantification of isomeric aminobutyric acids in human serum samples was followed the LC–MS/MS method published by our laboratory^14^. Briefly, mobile phases are methanol (A) and water containing 0.005% formic acid and 2.5 mM ammonium formate (B). The MS instrument was operated and optimized under positive electrospray (+ ESI) and multiple reaction monitoring modes (MRM). The *m/z* transitions (precursor to product ions) and their tuning voltages were selected from our published paper^14^ and further optimized based on the best MRM responses from instrumental method optimization software. All analyses and data processing were completed on Shimadzu LabSolutions V5.91 software (Shimadzu Scientific Instruments, Inc., Columbia, MD).

### Sample preparation for LC–MS/MS analysis

Ten microliter human serum samples and equal volume of IS mixture solution (1.2 µM, 0.1% formic acid in methanol, v/v) were added to 35 µL 0.1% (v/v) formic acid in methanol, followed by 20 min-shaking at room temperature and another 15 min-centrifugation at 15,000 × g, 4 °C to precipitate protein. The supernatant was directly transferred to an autosampler vial and 45 μL of each sample was injected for LC–MS/MS analysis.

The samples of standard calibration curves were prepared by spiking the pure standards in surrogate matrix 5% (w/v) BSA in PBS (pH7.4). The samples for ten-point calibration curves were prepared by diluting the working solution to 0.02–10.24 µM for GABA, and 0.08–81.92 µM for AABA. Then ten microliters of each standard sample were taken and treated following the same preparation procedures of serum samples for LC–MS/MS analysis.

### Statistical analysis

Data were summarized as mean ± SD. Normality was checked using Q-Q plot and Kolmogorov–Smirnov test. When there was evidence against normality, Kruskal–Wallis ANOVA or Wilcoxon rank sum test was also conducted, Supplementary Table 2. Otherwise, comparisons among groups were performed using Student’s t-test and one-way ANOVA with Tukey’s post-Hoc test (α = 0.05). Association analysis was performed using both Pearson (r) correlations and Spearman (ρ) correlations, while scatter plots were used to decide the appropriate correlations for interpretations, as Pearson correlations for linear associations and Spearman correlations for nonlinear but monotonic associations. To control for the effects of age and BMI, partial correlations were further calculated. With N = 120 as the total sample, N = 60 for female and male separately, and N = 20 for each sex/function combination, we had an 80% power at type I error level 0.05 to detect Pearson and Spearman correlations as 0.25 and 0.27, 0.35 and 0.37, and 0.58 and 0.61, respectively. SAS 9.4 (SAS Institute, Cary, NC, USA) was used for statistical analysis. Two-sided p-values < 0.05 were considered as significant.

## Results

### Quantification of GABA and AABA in human serum and correlations with age

**Figure 1:**
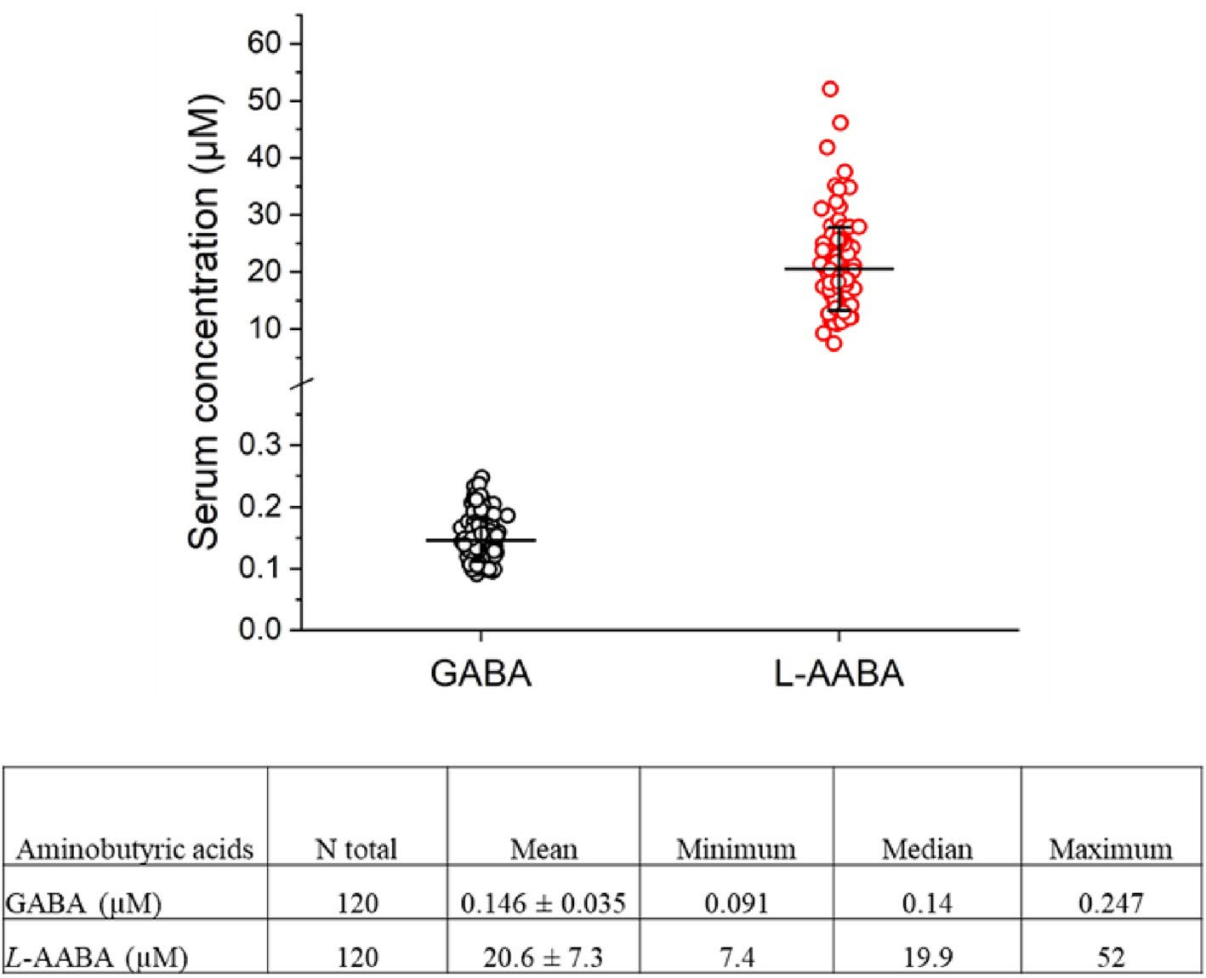
Comparison of serum levels of aminobutyric acids GABA and *L*-AABA. Figure legend: Comparison of serum levels of GABA and *L*-AABA in 120 white non-Hispanic individuals (aged 20–85 years). Mean ± SD, n = 120.

**Figure 2:**
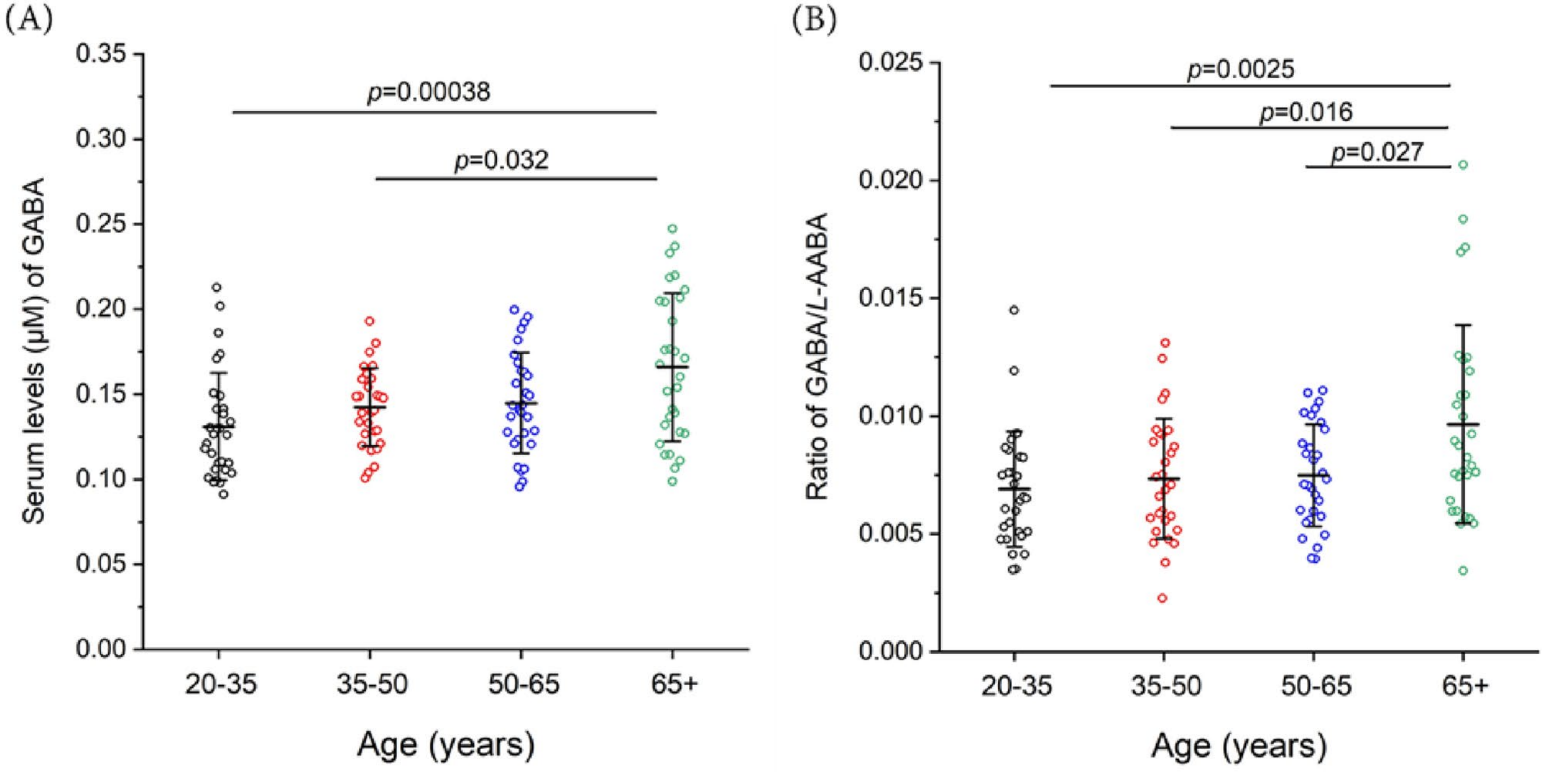
GABA concentration and G/A ratio in human serum from different age groups. Figure legend: (**A**) GABA, and (**B**) G/A ratios in human serum. n = 30 for each age group, mean ± SD. One-way ANOVA with Tukey post-Hoc test (α = 0.05).

Quantitation of the aminobutyric acids GABA and *L*-AABA from all 120 human samples gave concentrations of 0.146 ± 0.035 µM, and 20.6 ± 7.3 µM, respectively (Fig. 1). Concentrations of GABA and *L*-AABA, and the ratio of GABA to *L*-AABA levels (G/A ratio) are summarized in Table 2. Next serum levels were analyzed comparing age, sex, and physical parameters. Serum GABA levels significantly increased with age in the overall cohort (p = 0.0008, n = 120) and HP group (p = 0.0025, n = 40). The average serum levels of GABA in each age group were 0.131 µM (20–34 yrs), 0.142 µM (35–49 yrs), 0.145 µM (50–64 yrs), and 0.166 µM (65 yrs and up), respectively. These results indicate a substantial increase in the serum levels of GABA at age 65, which is significantly higher than that in the younger populations aged 20–34 yrs group (p = 0.00038) and 35–49 yrs group (p = 0.032) (Fig. 2A). Similar results were also obtained when calculating G/A ratio in serum. Average values of serum G/A ratio in different age groups were 0.0069 for 20–34 yrs, 0.0074 for 35–49 yrs, 0.0075 for 50–64 yrs, and 0.0097 for 65 yrs and up, respectively, and significant differences were also observed between the most senior group (age 65 +) and other younger age groups (p < 0.01, Fig. 2B). No significant sex-specific differences (p < 0.05) were observed (Table 2).

**Table 2:** Concentrations of aminobutyric acids GABA and *L*-AABA, and ratios of GABA/*L*-AABA (G/A ratio) in serum from different age or **sex** populations.

Physical performance	Aminobutyric acids	Whole cohort	Age group					Sex		
			20–34 yrs	35–49 yrs	50–64 yrs	65 + yrs	p-value	Female	Male	p-value
Overall	n	120	30	30	30	30		60	60	
	GABA (µM)	0.146 ± 0.035	0.131 ± 0.031	0.142 ± 0.023	0.145 ± 0.029	0.166 ± 0.043	0.0008***	0.150 ± 0.034	0.142 ± 0.035	0.248
	*L*-AABA (µM)	20.6 ± 7.3	20.5 ± 6.2	21.8 ± 8.8	20.8 ± 7.3	19.2 ± 6.7	0.589	20.7 ± 7.3	20.5 ± 7.3	0.879
	G/A ratio	0.0078 ± 0.0031	0.0069 ± 0.0024	0.0074 ± 0.0026	0.0075 ± 0.0022	0.0097 ± 0.0042	0.0021**	0.0079 ± 0.0030	0.0078 ± 0.0032	0.773
High (HP)	n	40	10	10	10	10		20	20	
	GABA (µM)	0.155 ± 0.040	0.139 ± 0.037	0.141 ± 0.021	0.147 ± 0.031	0.194 ± 0.044	0.0025**	0.158 ± 0.039	0.152 ± 0.041	0.653
	*L*-AABA (µM)	21.4 ± 8.5	21.8 ± 5.4	21.4 ± 11.4	21.7 ± 9.5	20.4 ± 8.0	0.984	22.2 ± 10.4	20.5 ± 6.2	0.519
	G/A ratio	0.0082 ± 0.0037	0.0066 ± 0.0017	0.0078 ± 0.0032	0.0075 ± 0.0025	0.0110 ± 0.0054	0.043*	0.0083 ± 0.0039	0.10082 ± 0.0036	0.904
Average (AP)	n	40	10	10	10	10		20	20	
	GABA (µM)	0.140 ± 0.034	0.128 ± 0.034	0.143 ± 0.022	0.143 ± 0.037	0.146 ± 0.043	0.675	0.150 ± 0.034	0.130 ± 0.032	0.063
	*L*-AABA (µM)	20.0 ± 5.7	17.5 ± 5.0	22.7 ± 6.5	19.7 ± 4.5	20.1 ± 6.1	0.230	19.7 ± 5.2	20.3 ± 6.2	0.737
	G/A ratio	0.0075 ± 0.0026	0.0079 ± 0.0031	0.0068 ± 0.0023	0.0075 ± 0.0021	0.0078 ± 0.0029	0.779	0.0081 ± 0.0026	0.0069 ± 0.0024	0.151
Low (LP)	n	40	10	10	10	10		20	20	
	GABA (µM)	0.143 ± 0.028	0.126 ± 0.024	0.143 ± 0.027	0.144 ± 0.022	0.158 ± 0.030	0.065	0.141 ± 0.026	0.145 ± 0.030	0.669
	*L*-AABA (µM)	20.4 ± 7.5	22.2 ± 7.4	21.2 ± 8.7	21.1 ± 7.6	17.0 ± 6.1	0.431	20.1 ± 5.1	20.6 ± 9.4	0.821
	G/A ratio	0.0078 ± 0.0029	0.0062 ± 0.0022	0.0074 ± 0.0022	0.0075 ± 0.0022	0.0102 ± 0.0036	0.012*	0.0075 ± 0.0022	0.0083 ± 0.0035	0.381

Each data represents mean ± SD. ANOVA was applied for statistics analysis. **p < 0.01, ***p < 0.001. For comparing female and male, the minimal detectable standardized mean differences at power level 80% and type I error level 0.05 were 0.5 for N = 60 per group and 0.9 for N = 20 per group. For comparisons among the four age groups, the minimal detectable effect sizes as the proportions of variations of the Y variable explained by the groups were 11% for N = 30 per group and 28% for N = 10 per group.

**Figure 3:**
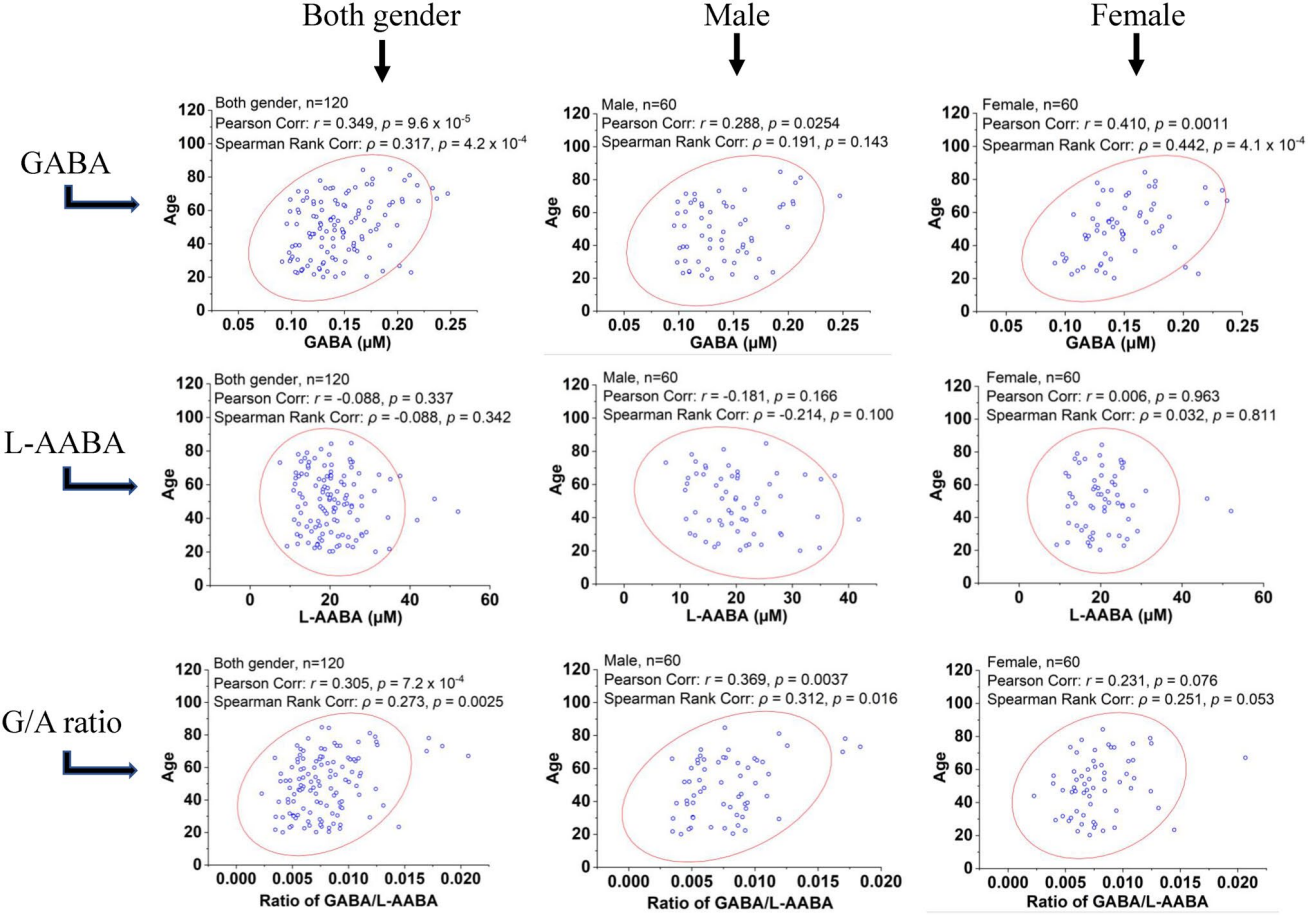
Correlations of serum levels of aminobutyric acids with age. Figure legend: Scatter plots with confidence ellipses (red line, 95% confidence level) were applied to virtually indicate the correlations between aminobutyric acids (GABA, *L*-AABA, and G/A ratio) and age in whole cohort (both sex), or male and female separately.

Next both Pearson and Spearman correlation tests were applied to investigate the associations of GABA and AABA serum levels with age in all 120 participants and on male and female populations (n = 60) separately. GABA showed a significant and positive association with age in the overall cohort (r = 0.349, ρ = 0.317, both p < 0.001). Furthermore, this association is stronger in females with increased correlation coefficients, but weaker in males with reduced correlation coefficients (Fig. 3). No significant association was obtained between serum *L*-AABA levels with age. The serum G/A ratio exhibited similar positive associations with age as GABA (Fig. 3). A significant and positive correlation was observed between serum G/A ratio and age in the overall cohort (r = 0.305, p = 0.00072; ρ = 0.273, p = 0.0025) and in males (r = 0.369, p = 0.0037; ρ = 0.312, p = 0.016), but it becomes much weaker in females (r = 0.231, p = 0.076; ρ = 0.251, p = 0.053).

### Significant correlations between aminobutyric acids with physical parameters and physical performance

All associations of GABA and AABA with p-values < 0.1 for physical performance and parameters are summarized in Table 3. Whereas GABA has positive association with age, it showed negative associations with physical performance (fast gait speed, 6MWT, PROMIS score, and SF36 PFS raw score) in the whole cohort. This suggests that elevated physical performance may be associated with reduced serum GABA levels. GABA levels in serum also negatively correlated with two BMD values (subtotal, and femoral neck) in the 120 human subjects. Furthermore, remarkable sex-related differences were observed when investigating the associations between GABA levels and physical characteristics and performance as GABA was only correlated negatively with femoral neck BMD in female subjects.

**Table 3:** Correlations of aminobutyric acids GABA and *L*-AABA with physical parameters and performance in 120 white non-Hispanic individuals (aged 20–85 years).

Sex	Parameters	n	GABA				*L*-AABA			
			Pearson		Spearman		Pearson		Spearman	
			r	p-value	ρ	p-value	r	p-value	ρ	p-value
Both	Age	120	0.3486	0.0001	0.3168	0.0004				
	Height	120	−0.2269	0.0127	−0.2537	0.0052				
	Usual gait speed	120					0.1566	0.0875		
	Fast gait speed	120	−0.1706	0.0625	−0.2078	0.0228				
	Six Min Distance	117			−0.1611	0.0828	0.2059	0.0259		
	Total SPPB score	120					0.1534	0.0944		
	PROMIS score	120			−0.1627	0.0759				
	SF36 PFS raw score	120			−0.2407	0.0081	0.1957	0.0322		
	Femoral neck BMD	117	−0.2175	0.0185	−0.2039	0.0275				
Female	Age	60	0.4100	0.0011	0.4419	0.0004				
	Femoral neck BMD	57	−0.2888	0.0294	−0.3051	0.0210				
Male	Age	60	0.2884	0.0254	0.2788	0.0815			−0.3289	0.0383
	BMI	60			0.2381	0.0669				
	Height	60	−0.3791	0.0028						
	Six Min Distance	58					0.3353	0.0101	0.2501	0.0583
	PROMIS score	60	−0.2398	0.0650	−0.2338	0.0723				
	SF36 PFS raw score	60			−0.3519	0.0058	0.2904	0.0244		
	Femoral Neck BMD	60					0.2291	0.0783	0.3214	0.0123

Pearson correlations (r) and Spearman Rank correlations (ρ) with p-value < 0.1 are shown in the Table.

As GABA significantly associated with age (Pearson r = 0.3486, p = 0.0001; Spearman ρ = 0.3168, p = 0.0004 Table 3, Supplementary Fig. 1), and it is well known that both age and BMI are highly associated with physical performance, BMD values, and lean/fat mass in both sexes (Supplementary Table 3), we further assessed these correlations after controlling the effect of age and/or BMI by partial correlation tests. The results summarized in Table 4, Supplementary Fig. 2 indicating that associations between serum GABA levels and physical performance scales, BMDs, and lean/fat mass values diminished in partial correlation tests in all physical capacity groups. Only two performance scales, best grip strength in the male HP group (Pearson and Spearman) and SF36 PFS raw score in male AP group (Pearson), showed negative associations with GABA levels after controlling the effects of age and BMI (Table 4, Supplementary Fig. 2).

**Table 4:** Correlations of the GABA in 120 white non-Hispanic individuals (aged 20–85 years) with different sex.

Pysical Capacity Group	Sex	Variable	N	Pearson Correlations								Spearman Rank Correlations					
				Ordinary		Partial on age			Partial on age, BMI			Ordinary		Partial on age		Partial on age, BMI	
				r	p-value	r'	p-value		r'		p-value	ρ	p-value	ρ'	p-value	ρ'	p-value
Overall	Male	Age	60	0.2884	0.0254	–	–		–		–			–	–	–	–
		BMI	60						–		–	0.2381	0.0669			–	–
		SF36 PFS raw score	60									−0.3519	0.0058				
		PROMIS score	60	−0.2396	0.0652							−0.2323	0.0741				
	Female	Age	60	0.4100	0.0011							0.4419	0.0004				
		Femoral neck BMD	57	−0.2888	0.0294							−0.3051	0.0210				
		Total Lean Mass	60													0.2561	0.0592
High (HP)	Male	Best Grip Strength	20	−0.5316	0.0157	−0.4540	0.0508		−0.5366		0.0217	−0.4197	0.0654	−0.4014	0.0885	−0.4478	0.0624
		PROMIS score	20	−0.5046	0.0233							−0.4214	0.0642				
		SF36 PFS raw score	20									−0.3919	0.0875				
		Total Lean Mass	20	−0.3868	0.0920				−0.4342		0.0718	−0.3985	0.0818			−0.4175	0.0847
		Total Fat Percentage	20	0.4115	0.0714												
	Female	Age	20	0.5426	0.0134	–	–		–		–	0.5594	0.0103	–	–	–	–
		Usual gait speed	20									−0.4778	0.0331				
		Fast gait speed	20	−0.4843	0.0305							−0.4756	0.0341				
		Time for 5 chair stands	20	0.4027	0.0784							0.4511	0.0459				
		RCS 30 s	20	−0.5030	0.0238							−0.4936	0.0270				
		Spinal BMD	19	−0.5836	0.0087	−0.4275	0.0768		−0.4181		0.0949	−0.4695	0.0425				
		Total Fat Mass	19						−0.4146		0.0980			−0.4483	0.0621	−0.5977	0.0113
Average (AP)	Male	Six Min Distance	19	−0.5418	0.0166	−0.5302	0.0236										
		Fast gait speed	20	−0.3952	0.0869												
		SF36 PFS raw score	20	−0.5722	0.0084	−0.5592	0.0128		−0.5040		0.0330						
		Total Lean Mass	20						−0.4633		0.0528					−0.4899	0.0391
		Total Fat Mass	20	0.4500	0.0465	0.4466	0.0553					0.5158	0.0199	0.5206	0.0223	0.5030	0.0333
	Female	Time for 5 chair stands	20									0.4045	0.0769				
		SF36 PFS raw score	20									−0.4118	0.0712				
Low (LP)	Female	Age	20	0.5999	0.0052	–	–		–		–	0.5940	0.0058	–	–	–	–
		PROMIS score	20									−0.4122	0.0709				
		Total Lean Mass	18					0.4303		0.0962							

Pearson correlations (r) and Spearman Rank correlations (ρ) with p-value < 0.1 are shown in the Table.

### Significant positive correlations between AABA with physical parameters and physical performance, but not with age

*L*-AABA also exhibited associations with physical performance, but unlike GABA, all associations of *L*-AABA are positive. *L*-AABA is positively associated with usual gait speed, 6MWT, total SPPB score, and SF36 PFS raw score in 120 human subjects, also with 6MWT and SF36 PFS raw score in the 60 male subjects, but no correlations were observed in the 60 female participants (Table 3, Supplementary Fig. 1). Interestingly, *L*-AABA showed more noteworthy sex-dependent differences in the individuals with high (HP) and low physical performance (LP) based on the results of physical performance assessment. Results summarized in Table 5 show that *L*-AABA positively associated with usual gait speed, fast gait speed, 6MWT, total SPPB score, and SF36 PFS raw score in 80 subjects from HP and LP groups, and stronger positive associations were found in males from both young (20–50 years old) and the aged (50–85 years old) populations. For example, associations (Pearson correlation) with 6MWT in all participants (HP and LP) is 0.236 (p = 0.038, n = 80) for both sexes and 0.387 (p = 0.015, n = 40) for males. In the young participants (HP and LP), associations with 6MWT are 0.290 (p = 0.069, n = 40) for both sexes and 0.439 (p = 0.053, n = 20) for males. In addition, almost no correlations between *L*-AABA and physical performance scales were observed in females in the HP group. All these results suggest that the serum levels of both GABA and *L*-AABA might be indicative of physical performance, but in opposite directions, which together could make them extremely useful biomarkers.

**Table 5:** Correlations of serum *L*-AABA levels in the combined extreme physical capacity groups–80 participants from both high and low performer groups, with physical parameters and performance scales.

Age group	Sex	N	Variable	Pearson		Spearman	
				r	p-value	ρ	p-value
All	Both	80	Total SPPB score	0.2232	0.0466		
		80	Usual gait speed	0.2550	0.0224	0.2740	0.0139
		80	Fast gait speed	0.1855	0.0995		
		78	Six Min Walk Test	0.2358	0.0377		
		80	SF36 PFS raw score	0.2244	0.0454		
	Male	40	Age			−0.3248	0.0409
		40	Total SPPB score	0.2684	0.0940		
		40	Usual gait speed	0.2683	0.0941	0.3218	0.0429
		39	Six Min Walk Test	0.3871	0.0149	0.3226	0.0452
		40	SF36 PFS raw score	0.3322	0.0363		
		40	Total % fat mass			−0.2984	0.0615
		40	Femoral neck BMD	0.3062	0.0547	0.3850	0.0142
Young (20–50 years)	Both	40	Six Min Walk Test	0.2902	0.0693		
		38	Spinal BMD	0.3207	0.0496	0.3342	0.0403
		38	Femoral neck BMD	0.3165	0.0529	0.3253	0.0462
	Male	20	Six Min Walk Test	0.4385	0.0531		
		20	Spinal BMD	0.4558	0.0434	0.4632	0.0397
		20	Femoral neck BMD	0.4048	0.0767	0.4556	0.0435
Aged (50–85 years)	Both	40	Usual gait speed	0.3968	0.0112	0.3070	0.0540
	Male	20	Usual gait speed			0.4283	0.0596
		20	SF36 PFS raw score	0.4155	0.0685		
		20	Appendicular lean/height^2^	0.4418	0.0511	0.4782	0.0330
	Female	20	Usual gait speed	0.4866	0.0296	0.4108	0.0720

Pearson correlations (r) and Spearman Rank correlations (ρ) with p-value < 0.1 are shown in the Table.

### Correlations between serum G/A ratio with physical parameters and performance

**Figure 4:**
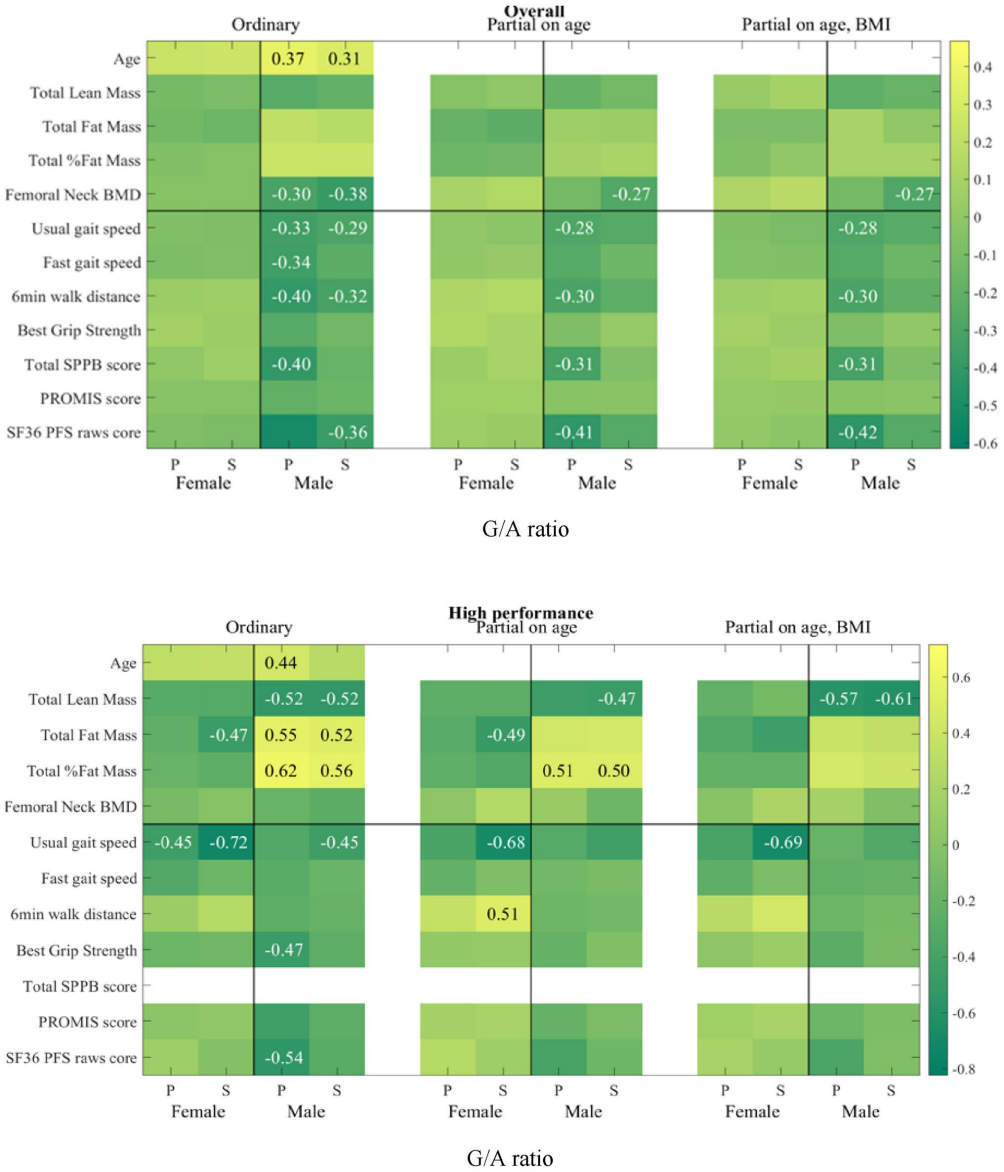

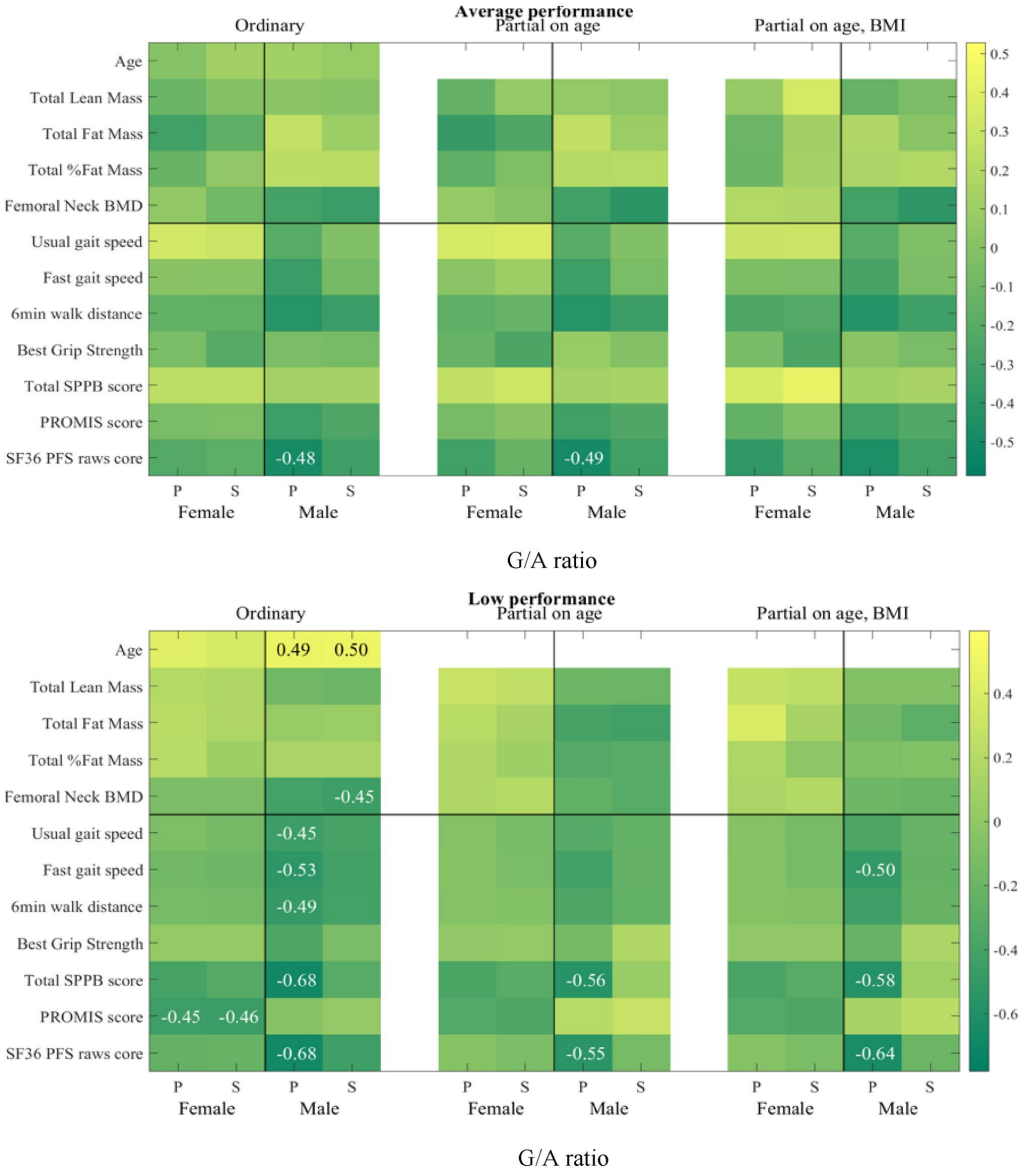
Heatmaps of correlations of G/A ratio. Figure legend: Heatmaps of correlations of G/A ratio in the 120 (overall) samples and in separate performing (HP, AP and LP) groups. P—Pearson correlations and S- Spearman correlations, only statistically significant correlations, p-values < 0.05, are shown.

As the associations of serum levels of GABA and *L*-AABA with human physical capacity were found to be opposite, we proposed that the ratio of GABA and *L*-AABA levels in serum could potentially serve as a more precise predictor of physical performance. Thus, we explored the relationships of serum G/A ratio with physical parameters in males and females separately, by using both Pearson and Spearman correlation tests (Table 6, Fig. 4). Serum G/A ratio in males was found to be negatively associated with 6 physical performance scales, including total SPPB score (r = −0.401, p = 0.002), usual gait speed (r = −0.334, p = 0.009), fast gait speed (r = −0.344, p = 0.007), 6MWT (r = −0.399, p = 0.002), and SF36 PFS raw score (r = −0.515, p < 0.0001). Additionally, serum G/A ratios negatively correlated with femoral neck BMD and total lean mass, and positively correlated with total fat percent in males (Table 6, Fig. 4). However, no associations were observed for serum G/A ratio with any physical performance and physical parameters but age and BMI in females. These results indicate that serum G/A ratio could be potentially linked to physical performance in the male population. Then we further performed partial Pearson and partial Spearman tests to evaluate the correlations of serum G/A ratio with physical parameters after controlling the effect of age and/or BMI. Pearson correlation results revealed that 5 out of 6 statistically significant associations without the covariate adjustments (ordinary) still have significantly negative associations after controlling the effect of age and age + BMI (partial) in the male population (Table 6, Fig. 4). All associations with BMD and lean/fat mass diminished greatly in partial correlation tests. This suggests that serum G/A ratios might negatively associate with physical performance in males, no matter the age and or metabolic status. A positive Spearman and Pearson association of G/A ratio with the total fat percentage (ρ 0.56, p 0.01, and r 0.616, p 0.004) and negative with the total lean mass (ρ −0.52, p = 0.02 and r −0.518, p = 0.019) was found in the subgroup of HP males, and still present after controlling the effect of age and age + BMI (partial). This group also had a negative Pearson correlation with the grip strength, PROMIS score and SF36 PFS raw score. The LP male group similarly showed a negative Pearson correlation with the physical parameters of fast gait speed, SF36 PFS raw score and total SPPB score. The HP female group had a negative Pearson and Spearman correlation with the usual gait speed and the LP female group with the PROMIS score.

**Table 6:** Correlations of the G/A ratio in 120 white non-Hispanic individuals (aged 20–85 years) with different sex.

Physical Capacity Group	Sex	Variable	N	Pearson Correlations						Spearman Rank Correlations					
				Ordinary		Partial on age		Partial on age, BMI		Ordinary		Partial on age		Partial on age, BMI	
				r	p-value	r'	p-value	r'	p-value	ρ	p-value	ρ'	p-value	ρ'	p-value
Overall	Male	Age	60	0.368	0.004					0.311	0.015				
		Total SPPB score	60	−0.401	0.002	−0.315	0.015	−0.315	0.016						
		Usual gait speed	60	−0.334	0.009	−0.275	0.035	−0.28	0.033	−0.291	0.024	−0.248	0.059	−0.242	0.067
		Fast gait speed	60	−0.344	0.007	−0.249	0.057	−0.25	0.059	−0.221	0.09				
		Best Grip Strength	60	−0.243	0.062										
		6 min walk test	58	−0.399	0.002	−0.299	0.024	−0.301	0.024	−0.317	0.015				
		SF36 PFS raw score	60	−0.515	< .0001	−0.41	0.001	−0.416	0.001	−0.364	0.004	−0.251	0.055	−0.247	0.062
		Femoral neck BMD	60	−0.303	0.019					−0.378	0.003	−0.267	0.041	−0.272	0.039
		Total Lean Mass	60	−0.24	0.065										
		Total Fat Percent	60	0.245	0.059					0.247	0.057				
	Female	Age	60	0.231	0.076					0.251	0.053				
		BMI	60							−0.224	0.085				
High (HP)	Male	Age	20	0.445	0.050										
		BMI	20							0.383	0.096				
		Usual gait speed	20							−0.449	0.047	−0.436	0.062		
		Best Grip Strength	20	−0.474	0.035										
		PROMIS score	20	−0.442	0.051										
		SF36 PFS raw score	20	−0.540	0.014	−0.391	0.098								
		Total Lean Mass	20	−0.518	0.019	−0.455	0.051	−0.573	0.013	−0.522	0.018	−0.467	0.044	−0.610	0.007
		Total Fat Mass	20	0.552	0.012	0.433	0.064			0.516	0.020	0.453	0.052		
		Total Fat Percent	20	0.616	0.004	0.506	0.027	0.456	0.058	0.559	0.010	0.500	0.029	0.405	0.095
	Female	Usual gait speed	20	−0.453	0.045					−0.723	0.000	−0.680	0.001	−0.687	0.002
		6-Min Walk Test	20									0.505	0.027	0.448	0.062
		Total Fat Mass	19							−0.467	0.044	−0.487	0.040	−0.455	0.067
		BMI	60							−0.224	0.085				
Average (AP)	Male	Six Min Distance	19	−0.413	0.079	−0.416	0.086	−0.418	0.095						
		SF36 PFS raw score	20	−0.479	0.033	−0.486	0.035	−0.457	0.057						
		Femoral neck BMD	20									−0.391	0.098		
Low (LP)	Male	Age	20	0.488	0.029					0.496	0.026				
		Total SPPB score	20	−0.680	0.001	−0.565	0.012	−0.575	0.013						
		Usual gait speed	20	−0.450	0.047					−0.394	0.086				
		Fast gait speed	20	−0.532	0.016	−0.420	0.073	−0.502	0.034	−0.423	0.063				
		Six Min Distance	20	−0.488	0.029			−0.433	0.073	−0.415	0.069				
		SF36 PFS raw score	20	−0.676	0.001	−0.547	0.015	−0.638	0.004	−0.435	0.055				
		Femoral neck BMD	20	−0.408	0.075					−0.448	0.048				
		Total Fat Mass	20			−0.395	0.094					−0.430	0.066		
	Female	Age	20	0.420	0.066										
		Total SPPB score	20	−0.400	0.081										
		PROMIS score	20	−0.445	0.049					−0.457	0.043				

Pearson correlations (r) and Spearman Rank correlations (ρ) with p-value < 0.1 are shown in the Table.

## Discussion

Aging muscle undergoes several morphological changes along with declining regenerative capacity, which affects muscle strength and physical performance^8^. As the inability to modulate corticospinal excitability has been linked to a declining motor performance with advancing age^25^, probing the roles of GABA, a main inhibitory neurotransmitter in brain, in age related musculoskeletal disorders is important and can lead to new therapeutic venues for these diseases, including sarcopenia. GABA is a highly conserved molecule from bacteria to humans with essential roles in all kingdoms^17,18,21^. It has a dual action on neurotransmission in the mammalian central nervous system via two distinct classes of GABA receptors, ionotropic GABA-A and metabotropic GABA-B receptors, which differ in their pharmacological, electrophysiological, and biochemical properties^31^^,^^26,27^. The majority of aging studies have focused on brain GABA levels^28,29^, while the associations between circulating GABA levels, age, and aging-related physical performance are seldom reported. Age related CNS GABA disorders are noteworthy and include: Parkinson’s disease, Alzheirmer’s disease, anxiety, depression, schiszophrenia, autism spectrum disorder, and others^30^.

Largely relegated to a third-tier in most studies is a group of all the diseases in GABA metabolism leading to spasticity disorders, including multiple sclerosis spasticity, cerebral pasly spasticity, tetanus spasticity, generalized dystonia, and even stiff man syndrome, which is a rare and disabling auto-immune neurologic disorder resulting in impairment of GABA function, characterized by muscle rigidity and episodic muscle spasms involving axial and limb musculurature. What has not been considered is that these diseases seem to have a strong peripheral component to their pathophysiology. In fact, that we can detect circulating GABA in plasma/serum indicates that it is acting peripherally and not just centrally. Obviously skeletal muscles will be a major target of these peripheral actions. We have reported that C2C12 muscle cells produce GABA^31^^,^^32^, another strong indication for peripheral actions of GABA in mammals. Although centrally produced GABA has been considered to have limited ability to cross the blood brain barrier, in humans it is feasible to assume that GABA measured in serum derives mainly from skeletal muscles.

In the 120 serum samples collected from healthy humans covering both sexes, from young to the elderly, and with different physical capacities, we demonstrated that GABA levels in the circulation are significantly associated with age. It increases consistently with aging in both males and females, however a positive association with physical activity was also observed but only in the female HP and female LP groups. The G/A ratio could be another good biomarker for aging with the same positive, but stronger correlations than GABA. Interestingly, when considering physical performance, a significant serum G/A ratio was only obtained in the male HP and male LP groups. This sex-related difference between GABA and the G/A ratio may come from* L*-AABA. Even though *L*-AABA levels did not show any associations with age in the whole cohort, it was found to be negatively correlated with age in HP and LP males. Taking both blood GABA levels and G/A ratios into consideration may significantly increase the strength of studies to discover more universal aging biomarkers not only in the general population but also specifically in males and females. It is now well known that traumatic injuries are more common in males, but females are more susceptible to and disproportionately affected by musculoskeletal conditions, many of these are age dependent such as osteoporosis^33^. It will be important to identify sex specific predictive and diagnostic biomarkers for musculoskeletal disorders and diseases.

Skeletal muscle physical performance is regulated by factors associated with the nervous, muscular, and skeletal systems. It has been well studied that muscle function is significantly related to the measures of BMD (femoral neck BMD, spine BMD, hip BMD, etc.) and body composition (fat percentage, lean mass, etc.)^34–36^. Aging-related muscle function loss is often accompanied by increased fat mass and percentage along with reduced lean mass and BMD. Our group has recently reported that two genes coding for the GABA receptors *GABBR2* and *GLRA1* and the gene coding for glutamate decarboxylase *GAD1*, showed an association with total body BMD, and that *GABBR2* is associated with bone fractures^14^. These genes are involved in both bone and muscle function and likely further influence overall musculoskeletal function and body metabolism^14^. In the present study, femoral neck BMD was found to be negatively correlated with GABA in the whole cohort and female group, and with G/A ratio in male group. When physical performance was taken into consideration, a strong negative association was observed between GABA and spine BMD in female HP, while the G/A ratio negatively associated with femoral neck BMD in male AP and LP groups. Moreover, serum GABA levels and G/A ratios also exhibited generally negative associations with lean mass and positive associations with fat mass/percentage in the different physical performance groups.

GABA levels were also negatively associated with various physical performance scales in our study, except for the time for 5 chair-stands. But this positive association with the chair-stand further confirmed that serum GABA concentrations increased when physical performance declines. This negative correlation between serum GABA levels and physical performance might be age-related, as the results of further partial association tests indicated that these correlations between GABA and performance scales diminished when controlling for the effects from age. All these findings suggest that aging is a key factor affecting GABA levels in circulation. This also could point to the peripheral actions of GABA, higher levels of GABA in muscles during aging might lead to substantial changes in muscle tonicity and even muscle spasms, which might contribute to the decline in muscle function during aging.

Even though no direct significant positive correlations were observed between GABA and physical performance assessments, GABA levels in the body are known to be affected by physical activity. Exercise can stimulate PGC1α-mediated GABA secretion from muscle^37^. Increased human activity through exercise has been shown to increase the levels of GABA in skeletal muscle (i.e., quadriceps), brain, and blood^38^, implying that GABA could work as an exercise/PGC1α-mediated myokine^37^. Our previous studies in 136 young to middle-aged healthy Caucasian females (aged 21–41 years) reported that serum GABA showed a positive association (ρ = 0.31, *p* = 0.0055) with physical activity (times per week) in lean females (BMI 18.5–24.9 kg m^−2^), but no association in obese females with BMI ≥ 25.0 kg m^−2^^14^. This might be caused by different metabolomic pathways for GABA in muscle and adipose tissues, indicating that metabolic homeostasis (anabolic/catabolic balance, hormone/protein/amino acid deficiencies, etc.) during aging may affect GABA levels in the circulation.

Unlike GABA levels, the associations between G/A ratio with physical performance assessments appear to be more independent from age. Particularly in the 60 male participants, 5 out of 6 negative associations with various physical performance tests of mobility (total SPPB score, usual gait speed, fast gait speed, 6 min walk test, and SF36 PFS raw score) were still significant after controlling for the effect of age using partial Pearson correlation analysis. SF36 PFS raw scores also showed medium to strong correlations (r-values ranged from −0.4 to −0.6) with serum GABA levels in all three male HP, AP and LP groups. The short Form-36 Physical Functioning Scale (SF36 PFS) questionnaire is a set of patient self-reporting measures to indicate health status. The SF-36 PFS is composed of 10 items encompassing a hierarchical range of difficulties on everyday activities, including vigorous activities, moderate activities, lifting/carrying, climbing stairs, bending/kneeling/stooping, and walking, etc., and it has been used as a reliable and valid “stand-alone” instrument in research describing activity limitations in the elderly living in the community, and patients with different minor pathophysiological conditions and chronic diseases^39–41^. These results strongly suggest that G/A ratio could be a potential circulating biomarker to indicate change in physical performance, particularly mobility performance in males.

The current study possesses limitations that require consideration when interpreting the data. The study is cross-sectional in nature such that causal relationships between the proposed biomarkers and physical activity/performance remain unclear. It is unknown if altered muscle status (mass, strength, power, fatigue, etc.) is responsible for or is affected by the levels of biological factors in the circulation. However, the significant associations we observed with age suggest that serum GABA levels and the G/A ratio in humans may be related to aging processes as their associations are all negative. A limitation of the current study is its inclusion of white, non-Hispanic participants. Further exploration is required to determine whether the findings translate to other races and ethnic groups. Similarly, sex appears to be a driver of the utility of these biomarkers which cannot be fully explored with the current data.

## Conclusions

Together, these observations suggest that serum aminobutyric acids, especially GABA and G/A ratio, are closely associated with aging-related physical performance. These studies also highlight the potential role of GABA acting in peripheral tissues and its important contributions to muscle tonicity and muscle spasms. As both intrinsic (inflammation, apoptosis, mitochondria, calcium metabolism, etc.) and extrinsic (endocrine, nutritional status, immobility, etc.) factors contribute to progressively defective myogenesis and muscle dysfunction during aging, considering multiple serum biomarkers may more accurately predict the correlations between aging, physical performance, and the beneficial effects from exercise. These studies could also open up new therapeutical venues for muscle disorders such as stiff man syndrome and sarcopenia.

### Supplementary Information

Supplementary Information.

## Data Availability

All data generated and analyzed during this study are included in this published article and its supplementary information files.

## Abbreviations

6MWT: Six-minute walk test
AABA: α-Aminobutyric acid
BMD: Bone mineral density
BMI: Body mass index
BSA: Bovine serum albumin
ESI: Electrospray ionization
GABA: γ-Aminobutyric acid
IS: Internal standard
LC: Liquid chromatography
LC–MS/MS: Liquid chromatography–tandem mass spectrometry
MRM: Multiple reaction monitoring
MS: Mass spectrometry
m/z: Mass-to-charge ratio
PBS: Phosphate-buffered saline
MSK: Musculoskeletal
SD: Standard deviation
