# Supplementary material for: γ-Aminobutyric acids (GABA) and serum GABA/AABA (G/A) ratio as potential biomarkers of physical performance and aging
Source: Sci Rep. 2023 Oct 10;13:17083. doi: 10.1038/s41598-023-41628-x (PMC10564855; doi:10.1038/s41598-023-41628-x)
Supplement: Supplementary file 1 — Supplementary Information. [file 41598_2023_41628_MOESM1_ESM.pdf]

γ-Aminobutyric acids (GABA) and serum GABA/AABA (G/A) ratio as potential biomarkers of physical performance and aging.

Charalampos Lyssikatos MD[1,†], Zhiying Wang PhD[2,†], Ziyue Liu PhD, BMED[1,3], Stuart J. Warden PT, PhD, FACSM, FASBMR[1,4], Lynda Bonewald PhD[1], Marco Brotto, BSN, MPharm, PhD[2*]

Supplementary Data

Supplementary Table 1. Demographics and Characteristics of 120 white non-Hispanic individuals (aged 20–85 years) recruited from the ICMH-Clinical Research Center FitCore Program.

	Parameters	N total	Mean	SD	Median	Range	
						Minimum	Maximum
Characteristics of participants	Age (years)	120	49.60	17.58	50.08	20.09	84.73
	Height (cm)	120	170.70	8.91	169.55	149.00	191.80
	Weight (kg)	120	78.13	13.62	77.80	54.10	114.10
	BMI (kg/m^2)	120	26.83	4.48	25.55	19.70	45.10
	Appendicular lean mass per height (ALM/height, kg/m)	117	7.84	1.39	7.98	4.64	12.14
	Whole body BMD (g/cm^2)	116	1.11	0.15	1.11	0.77	1.43
	Spine BMD (g/cm^2)	117	1.09	0.17	1.09	0.68	1.45
	Femoral Neck BMD (g/cm^2)	117	0.89	0.17	0.85	0.55	1.35
Physical performance scales	Total SPPB score	120	11.65	0.88	12.00	7.00	12.00
	SPPB gait speed score	120	3.99	0.09	4.00	3.00	4.00
	Usual gait speed (m/s)	120	1.40	0.18	1.40	0.74	2.00
	Fast gait speed (m/s)	120	2.05	0.34	2.05	1.01	3.28
	Best Grip Strength (kg)	120	34.53	12.80	34.85	6.60	63.00
	Time for 5 chair stands (s)	120	8.87	2.96	8.08	2.13	19.77
	6-min walk test (m)	117	569.62	109.81	580.00	160.00	969.00
	PROMIS score	120	56.42	7.27	54.70	40.40	73.30
	SF-36 PFS raw score	120	92.96	12.79	100.00	20.00	100.00

Supplementary Table 2. Normality test by Kolmogorov-Smirnov test, p values

By age groups, if non-normal, Kruskal-Wallis ANOVA

Function	Variable	20-34	35-49	50-64	65+	KW ANOVA
All	GABA	0.13	>0.15	>0.15	>0.15	
	L-AABA	>0.15	0.02	0.03	0.10	0.73
	G/A	>0.15	>0.15	>0.15	0.14	
High	GABA	>0.15	>0.15	>0.15	>0.15	
	L-AABA	>0.15	0.01	0.04	>0.15	0.72
	G/A	>0.15	>0.15	>0.15	>0.15	
Average	GABA	0.11	>0.15	0.12	>0.15	
	L-AABA	>0.15	>0.15	>0.15	>0.15	
	G/A	>0.15	>0.15	>0.15	>0.15	
Low	GABA	>0.15	>0.15	>0.15	>0.15	
	L-AABA	>0.15	>0.15	>0.15	>0.15	
	G/A	>0.15	>0.15	>0.15	>0.15	

By gender, if non-normal then Wilcoxon rank sum

Function	Variable	Female	Male	Wilcoxon
All	GABA	0.07	0.03	0.17
	L-AABA	0.01	0.06	0.76
	G/A	0.01	0.02	0.60
High	GABA	>0.15	>0.15	
	L-AABA	0.01	>0.15	0.84
	G/A	>0.15	0.03	0.78
Average	GABA	>0.15	0.01	0.05
	L-AABA	>0.15	>0.15	
	G/A	0.11	0.06	
Low	GABA	>0.15	>0.15	
	L-AABA	>0.15	>0.15	
	G/A	>0.15	>0.15	

Supplementary Table 3. Pearson and Spearman correlations for age/BMI with different physical performance scales, BMD values, and lean/fat mass in humans.

			Pearson Correlations										Spearman Rank Correlations									
			Female					Male					Female					Male				
		Physical performance levels		Age		BMI		N	Age		BMI		N	Age		BMI		N	Age		BMI	
	Variables		N	r	p-value	r	p-value		r	p-value	r	p-value		ρ	p-value	ρ	p-value		ρ	p-value	ρ	p-value
Physical performance scales	Total SPPB score	Overall	60					60	-0.3410	0.0077			60					60	-0.3449	0.0070		
		HP	20					20					20					20				
		AP	20	-0.3969	0.0831			20					20					20				
		LP	20					20	-0.5467	0.0126			20					20	-0.6754	0.0011		
	Usual gait speed	Overall	60	-0.2486	0.0554	-0.2457	0.0585	60	-0.2316	0.0749	-0.2624	0.0428	60	-0.2345	0.0713	-0.2780	0.0315	60				
		HP	20	-0.3944	0.0853			20			-0.5913	0.0060	20	-0.4018	0.0791			20			-0.5158	0.0199
		AP	20					20					20					20				
		LP	20					20	-0.3899	0.0892			20					20	-0.4039	0.0774		
	SPPB gait speed score	Overall	60	.	.	.	.	60					60	.	.	.	.	60				
		HP	20	.	.	.	.	20					20	.	.	.	.	20				
		AP	20	.	.	.	.	20					20	.	.	.	.	20				
		LP	20	.	.	.	.	20					20	.	.	.	.	20				
	Fast gait speed	Overall	60	-0.3779	0.0029	-0.2709	0.0363	60	-0.3426	0.0074			60	-0.3848	0.0024	-0.3414	0.0076	60	-0.2998	0.0199		
		HP	20	-0.3973	0.0828			20					20					20				
		AP	20	-0.5843	0.0068			20					20	-0.6138	0.0040	-0.5121	0.0210	20				
		LP	20					20	-0.4027	0.0784			20					20	-0.4808	0.0319		
	Best Grip Strength	Overall	60	-0.2290	0.0784	-0.2712	0.0361	60	-0.4865	<.0001			60	-0.2420	0.0625	-0.2865	0.0265	60	-0.4736	0.0001		
		HP	20	-0.6538	0.0018			20	-0.8234	<.0001			20	-0.5950	0.0057			20	-0.8071	<.0001		
		AP	20	-0.9287	<.0001			20	-0.7926	<.0001			20	-0.9214	<.0001			20	-0.7695	<.0001		
		LP	20					20	-0.5214	0.0184	-0.4339	0.0559	20			-0.3857	0.0931	20	-0.4842	0.0305		
	Time for 5 chair stands	Overall	60	0.2167	0.0964			60	0.2890	0.0251			60	0.2321	0.0744	0.2372	0.0680	60	0.2226	0.0874		
		HP	20					20	0.4782	0.0329			20	0.4105	0.0722			20	0.4355	0.0549		
		AP	20	0.6797	0.0010			20					20	0.6662	0.0013			20				
		LP	20					20	0.4004	0.0802			20					20	0.4301	0.0584		
	RCS 30s	Overall	60	-0.2931	0.0230	-0.2165	0.0966	60	-0.3028	0.0187			60	-0.2878	0.0258	-0.2433	0.0610	60	-0.2910	0.0241		
		HP	20	-0.5277	0.0168			20	-0.5376	0.0145			20	-0.6012	0.0050			20	-0.5884	0.0064		
		AP	20	-0.8209	<.0001			20	-0.5602	0.0102			20	-0.8326	<.0001			20	-0.6623	0.0015		
		LP	20					20					20					20	-0.3800	0.0984		
	6 min walk test	Overall	59	-0.3053	0.0187	-0.3288	0.0110	58	-0.4509	0.0004	-0.3290	0.0117	59	-0.3011	0.0205	-0.3433	0.0078	58	-0.4570	0.0003	-0.3035	0.0205
		HP	20	-0.5211	0.0185	-0.4498	0.0466	19	-0.4631	0.0459			20	-0.4331	0.0565			19	-0.5088	0.0261		
		AP	20					19	-0.5639	0.0119	-0.6478	0.0027	20					19	-0.4860	0.0349	-0.5645	0.0118
		LP	19					20	-0.4062	0.0755			19					20	-0.4541	0.0443		
	PROMIS score	Overall	60	-0.3830	0.0025			60	-0.4693	0.0002	-0.2829	0.0285	60	-0.3877	0.0022			60	-0.4240	0.0007	-0.2383	0.0667
		HP	20	-0.3814	0.0971			20	-0.6848	0.0009			20					20	-0.6898	0.0008		
		AP	20	-0.4326	0.0568			20	-0.4271	0.0603			20	-0.4005	0.0802			20				
		LP	20	-0.4087	0.0736			20	-0.4422	0.0509	-0.4645	0.0391	20	-0.4144	0.0692			20	-0.4155	0.0685	-0.4164	0.0678
	SF36 PFS raw score	Overall	60	-0.5103	<.0001	-0.2172	0.0955	60	-0.5011	<.0001	-0.3132	0.0148	60	-0.4905	<.0001	-0.2212	0.0894	60	-0.5087	<.0001	-0.2604	0.0445
		HP	20			-0.5478	0.0124	20	-0.5636	0.0097			20	-0.3812	0.0973			20	-0.5669	0.0091	-0.4378	0.0535
		AP	20	-0.6831	0.0009			20	-0.4827	0.0311			20	-0.7353	0.0002			20	-0.3840	0.0946		
		LP	20	-0.4552	0.0437			20	-0.6112	0.0042	-0.4448	0.0494	20					20	-0.7080	0.0005	-0.5080	0.0222

**BMD values**	**Total BMD**	**Overall**	56	-0.2961	0.0267	0.3071	0.0213	60	-0.3989	0.0016			56	-0.2545	0.0583	0.3413	0.0101	60	-0.4132	0.0010		
		**HP**	18	-0.6507	0.0035			20	-0.4174	0.0671			18	-0.4781	0.0448			20	-0.3949	0.0849		
		**AP**	20					20					20					20				
		**LP**	18			0.4620	0.0536	20	-0.4238	0.0626			18			0.5196	0.0271	20	-0.5243	0.0176		
	**Spine BMD**	**Overall**	57	-0.2357	0.0775			60			0.2168	0.0961	57	-0.2554	0.0552			60				
		**HP**	19	-0.5703	0.0108			20					19	-0.5450	0.0158			20				
		**AP**	20					20					20					20				
		**LP**	18					20			0.3996	0.0809	18					20				
	**Femoral Neck BMD**	**Overall**	57	-0.6350	<.0001			60	-0.5738	<.0001			57	-0.6843	<.0001			60	-0.5113	<.0001		
		**HP**	19	-0.6550	0.0023			20	-0.5835	0.0069	-0.3794	0.0990	19	-0.7000	0.0008			20	-0.4453	0.0491	-0.4304	0.0582
		**AP**	20	-0.6890	0.0008			20	-0.7356	0.0002			20	-0.7368	0.0002			20	-0.6807	0.0010		
		**LP**	18	-0.5720	0.0131			20	-0.4354	0.0550			18	-0.6409	0.0042			20	-0.4105	0.0722		
**Lean/fat mass**	**Total Lean Mass**	**Overall**	57	-0.3639	0.0054	0.3643	0.0053	60			0.3563	0.0052	57	-0.3830	0.0033	0.3360	0.0106	60	-0.2604	0.0445	0.3342	0.0091
		**HP**	19			0.6121	0.0053	20					19			0.6655	0.0019	20				
		**AP**	20	-0.5918	0.0060			20			0.7306	0.0003	20	-0.5383	0.0143			20			0.6629	0.0014
		**LP**	18			0.4585	0.0556	20					18			0.4407	0.0672	20				
	**Total Fat Mass**	**Overall**	57	0.2381	0.0745	0.8731	<.0001	60	0.3956	0.0018	0.8537	<.0001	57	0.2482	0.0627	0.8316	<.0001	60	0.3938	0.0019	0.8370	<.0001
		**HP**	19			0.8485	<.0001	20	0.4741	0.0347	0.8191	<.0001	19			0.7234	0.0005	20	0.4331	0.0565	0.7845	<.0001
		**AP**	20	0.5002	0.0247	0.8394	<.0001	20			0.8090	<.0001	20	0.4090	0.0733	0.8402	<.0001	20			0.9082	<.0001
		**LP**	18			0.9553	<.0001	20	0.6268	0.0031	0.9304	<.0001	18			0.9298	<.0001	20	0.6632	0.0014	0.8528	<.0001
	**Total Fat Percent**	**Overall**	57	0.3822	0.0033	0.6147	<.0001	60	0.4764	0.0001	0.5639	<.0001	57	0.3534	0.0070	0.6596	<.0001	60	0.5062	<.0001	0.6682	<.0001
		**HP**	19			0.7337	0.0004	20	0.5095	0.0218	0.6979	0.0006	19			0.6037	0.0062	20	0.5122	0.0209	0.7359	0.0002
		**AP**	20	0.6743	0.0011			20					20	0.5744	0.0081	0.5175	0.0194	20			0.4650	0.0388
		**LP**	18			0.8787	<.0001	20	0.7046	0.0005	0.8374	<.0001	18			0.9195	<.0001	20	0.6702	0.0012	0.8614	<.0001

Pearson correlations (r) and Spearman Rank correlations (ρ) with p-value < 0.1 are shown in the Table.

Supplementary Figure 1. Heatmaps of correlations of the GABA and L-AABA in 120 white non-Hispanic individuals (aged 20–85 years) with different sex. P - Pearson correlations and S- Spearman correlations, only statistically significant correlations, p-values <0.05, are shown.

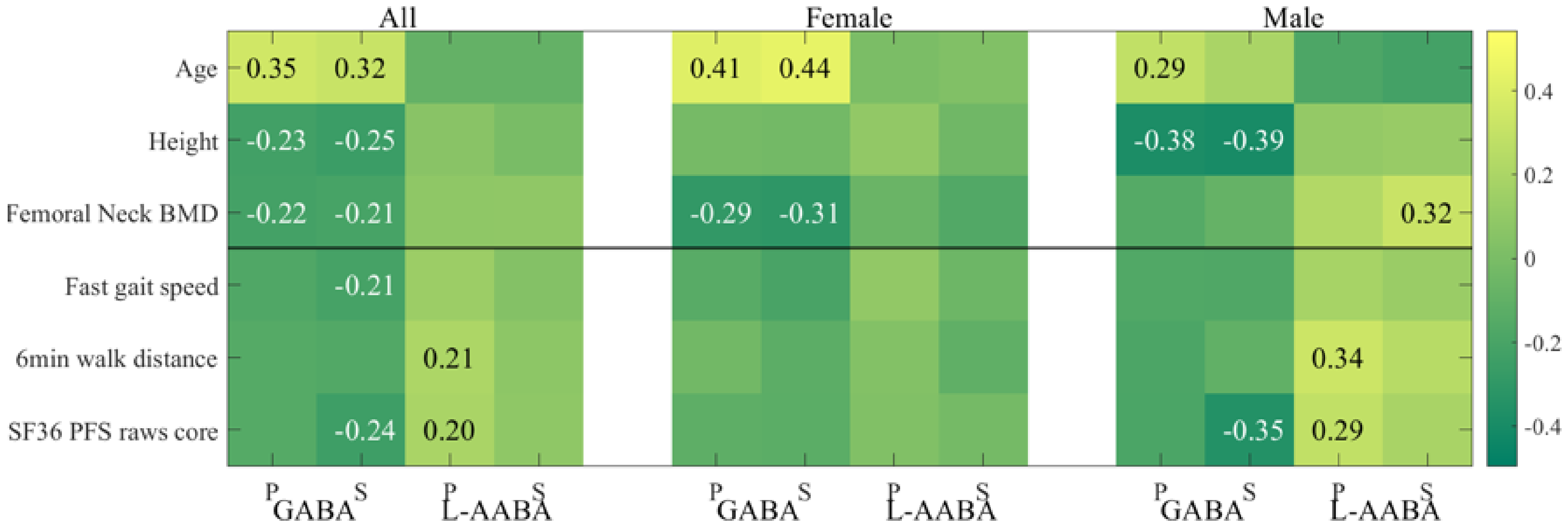

Supplementary Figure 2. Heatmaps of GABA associations with age and after controlling the effects of age and BMI, in overall (120 samples), HP, AP and LP groups. P - Pearson correlations and S- Spearman correlations, only statistically significant correlations, p-values <0.05, are shown

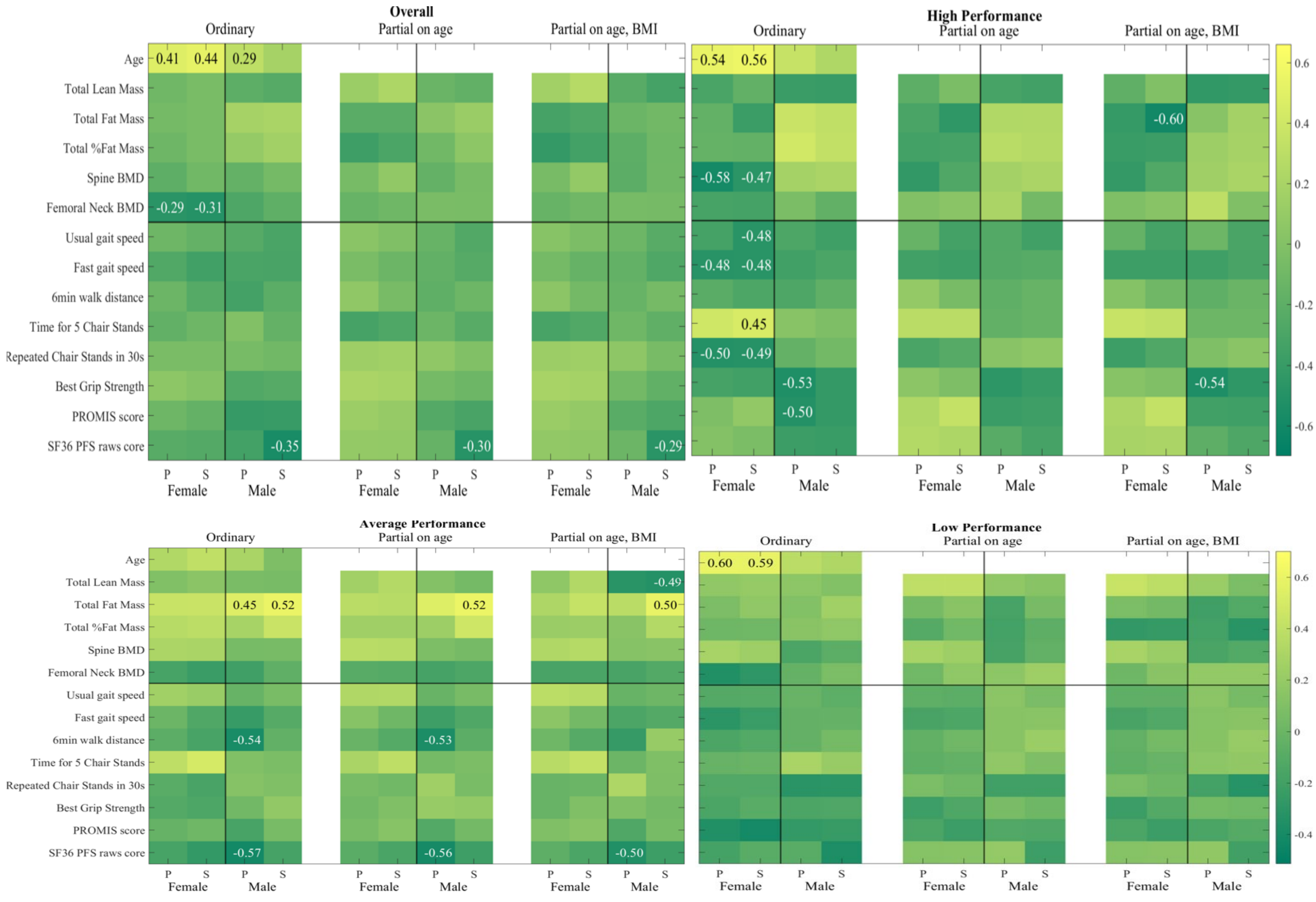
Overall
Ordinary
Partial on age
Partial on age, BMI
High Performance
Average Performance
Low Performance
Age
Total Lean Mass
Total Fat Mass
Total %Fat Mass
Spine BMD
Femoral Neck BMD
Usual gait speed
Fast gait speed
6min walk distance
Time for 5 Chair Stands
Repeated Chair Stands in 30s
Best Grip Strength
PROMIS score
SF36 PFS raws core
P
S
Female
Male
0.6
0.4
0.2
0
-0.2
-0.4
-0.6

GABA
